# Influence of COVID-19 pandemic lockdown on a sample of Egyptian children with Down syndrome

**DOI:** 10.1186/s43042-022-00280-2

**Published:** 2022-03-16

**Authors:** Nagwa A. Meguid, Neveen Hassan Nashaat, Hanaa Reyad Abdallah, Maha Hemimi, Ahmed Elnahry, Hazem Mohamed El-Hariri, Amal Elsaeid

**Affiliations:** 1 Research on Children with Special Needs Department, Medical Research Division, National Research Centre, Elbuhouth Street, Dokki 12622 Cairo, Egypt; 2 Biological Anthropology Department, Medical Research Division, National Research Centre, Cairo, Egypt; 3 Community Medicine Department, Medical Research Division, National Research Centre, Cairo, Egypt

**Keywords:** COVID-19, Down syndrome, Cognition, Language, Motor, Intervention

## Abstract

**Background:**

Down syndrome (DS) is characterized by variable degrees of intellectual disability (ID). The coronavirus disease-2019 (COVID-19) lockdown prevented children with DS from reaching their rehabilitation facilities. This could have led to deterioration of their abilities and mental health hazards. The aim of this cohort study was to investigate frequency of COVID-19, the influence of COVID-19 pandemic on health, and some abilities of children with DS, and to explore factors that could have governed receiving home-based training during the lockdown. A survey of 150 individuals with Down syndrome was answered by their caregivers. Additionally, 135 participants were subjected to assessment of cognitive, language, and motor abilities using Portage program. They were divided into 2 groups: group I who received online therapy sessions during the lockdown and group II who did not receive sessions. Logistic regression was used to determine the factors which influenced getting home-based training.

**Results:**

The percentage of COVID-19 cases was 3.3%. All evaluated abilities were reduced despite receiving online sessions particularly language performance (*P* < 0.001). Male gender, having severe ID and low parental education were among the factors which encouraged parents to get virtual training.

**Conclusion:**

COVID-19 pandemic had a negative impact on the abilities of DS children even those who got rehabilitation sessions. Their dependence on social interaction could have limited the benefit of virtual sessions. Factors that influence a parent’s decision to get home-based training should be monitored and targeted in order to overcome obstacles or concepts that may prevent families from enduring home-based intervention.

## Background

Down syndrome (trisomy 21) is a genetic chromosomal disorder that results from extra genetic material on chromosome 21. The frequency of Down syndrome (DS) has been estimated to be 1 in 700 births. It is the most common cause of intellectual disability (ID) attributed to chromosomal abnormalities [1]. Individuals with DS manifest immunological dysregulation. This could be attributed to overexpression of genes responsible for immunity on chromosome 21, such as those that code for certain interferon receptors [2]. These receptors are known to be influenced by some interleukins such as IL-10 and IL-22. The body cells of DS population are hypersensitive to interferon stimulation. Furthermore, T-cells were reported to be activated in the absence of clear infection [3]. Individuals with trisomy 21 were reported to have a relatively high risk of developing more severe symptoms of Coronavirus disease -19 (COVID-19) and secondary bacterial infection with high rates of hospitalization, admission to intensive care, and mortality due to severe acute respiratory syndrome coronavirus 2 (SARS-CoV-2) infection [4].

In addition to the particular physical features which make them get the diagnosis mostly since birth, individuals with DS manifest cognitive, language, and motor developmental delay. The cognitive abilities of such children showed mostly deficits in all types of verbal memory. Individuals with DS manifest morpho-syntactic and phonological deficits which make expressive language performance more delayed than receptive language performance. Children with DS manifest delay in the development of gross and fine motor abilities [5]. Early intervention has been found to result in adequate abilities performance of such cases in the future. Those who do not receive early regular rehabilitation, especially of cognitive, linguistic, and motor abilities may face greater difficulties in the future [6]. Encouraging the parents to participate in interventional programs is critical for the rehabilitation process and leads to improved performance of DS children [7]. Many countries, including Egypt, have been put on lockdown since World Health Organization (WHO) declared COVID-19 a pandemic in March 2020. Many individuals with DS have been unable to receive effective rehabilitation and comprehensive treatment in health facilities as a result of the lockdown, which could have a significant negative impact on their developmental abilities [4].

Egypt is a developing country with overcrowding reported along the Nile’s banks, in the Delta, and in major cities such as Cairo. This would lead to high risk of catching infection. Furthermore, the pandemic lockdown has been linked to mental health hazards [8]. Therefore, the aim of this study was to explore the influence of the COVID-19 pandemic lockdown on the cognitive, language, and motor abilities of a group of children with Down syndrome and to determine the health hazards and the frequency of COVID-19 among a sample of Egyptian individuals with DS. Factors that could have governed getting home-based training were also investigated.

## Methods

This study was a cohort prospective study and included a survey part. The participants were 150 individuals with Down syndrome (age range: 8 months–15 years; 72 males and 78 females). They visited the outpatient clinic of the research on children with special needs department, Medical Research Centre of Excellence, National Research Centre, Cairo, Egypt. The inclusion criteria were the cases with Down syndrome who visited the clinic before the COVID-19 lockdown and a chronological age more than 3 months before the lockdown in March 2020. Exclusion criteria were absence of baseline assessment data prior to the lockdown. A health survey was used to assess the health status and COVID-19 prevalence among participants. The health survey included 25 questions which covered personal data, health status of the proband, and COVID-19 related symptoms, test, and treatment. Moreover, questions investigating the influence of the lockdown on the abilities of the participants according to the parents’ subjective opinion were included [9]. For the objective assessment of the participants’ abilities, the participants who were between 3 months and 6 years (*N* = 135) and their abilities were evaluated in February 2020 before the lockdown were reevaluated after the end of the lockdown in August 2020. They were subjected to evaluation of their cognitive, language, and motor abilities by Portage program [10] alongside the routine clinical examination. The portage scores calculation depends on the relation between the scores the child obtained and the scores which are expected for his or her chronological age. Anthropometric assessment of weight, height, and head circumference using standardized equipment was also done to judge the health status of the participants. Bodyweight (kg) was assessed when the child was in light clothes and height (cm) was measured when the child was barefoot and knees stretched. All parameters were recorded to the nearest 0.1 value. All these measurements followed the method of international biological program. The Z scores for weight, height, and head circumference were calculated using the WHO Child Growth Anthro Plus program [11]. These 135 DS cases were divided into two groups: Group I who received home-based training by virtual online sessions during the lockdown and group II who did not receive sessions during the lockdown. The online sessions included instructions to the care giver (mostly the mother) regarding how to train the child to acquire the targeted abilities. The session lasted for 45 min. It was instructed by an experienced health professional. The session plan was created using the results of the most recent Portage assessment of the child's skills. The mother was given a chance to apply what she was instructed to do with her child to ensure that she got the session’s goals. If the child was alert enough to pay attention to the health professional on the screen, the session was given to the youngster himself with the caregiver. A list of the materials used in the session was sent to the caregiver one week before the session when the parents have to provide them. Other materials such as pictures were provided to the parents during the session. The rate of sessions was 2 sessions/week. During the session, cognitive, language, and motors abilities were targeted for enhancement. For example: improving visual matching skills, adaptive behavior, and interaction with others; activities for building up receptive and expressive vocabulary, sentences, and syntax with dolls, in kitchen or all around the house; correction of phonological processes or errors; expanding auditory and visual memory capacity; building up reading, writing, math and spelling abilities; activities to strengthen eye-motor coordination; enhancing gross and fine motor performance. Written informed consents were obtained from the participants’ parents. The study was approved by the Medical Research Ethics Committee of the National Research Centre.

### Statistical methods

The collected data were coded, tabulated then statistically analyzed using SPSS (Statistical Package for Social Sciences) statistics software version 22.0, IBM Corp., Chicago, USA, 2013 and Microsoft Office Excel 2007. Descriptive statistics were done for quantitative data as mean ± SD (standard deviation) and number-percentage for qualitative data. Inferential analysis was performed for quantitative variables using independent t-test and qualitative data using Chi-square test and Fisher’s Exact test for variables with small expected numbers with post hoc Bonferroni test for multiple comparisons. Correlations were tested by Spearman correlation coefficient. Logistic regression was used to determine factors affecting performing home training. The level of significance was taken at *P* value < 0.050.

## Results

### Results of the health survey

The data of age of the participants, their gender, and the gender of their caregiver are presented in Table 1. Most of the participants were from Giza governorate (~ 34%), followed by Cairo (~ 26%). The remaining participants were from other governorates in upper and lower Egypt such as Menia, Qalioubiya, Sharqiya, Alexandria. Lower percentage were from Fayyoum, Bani Suef, Suez, Assiut, Gharbiya, Loxor, and Port Said. All the participants (*N* = 150) had separate extra copy of chromosome 21 (47, XY, or XX) except 2 cases who manifested translocation type. The subjective degree of ID ranged from mild to severe and 14% reported no ID. All participants lived with their families. About 62% reported no comorbidities. The number of comorbidities in 48% of participants ranged from 1 to 3. They were mostly congenital heart disease, vitiligo, hypothyroidism, or excessive hair loss. About 15% manifested short stature (*N* = 23). No one had *Z* score less than (− 2.5) regarding body weight. Microcephaly was reported in about 20% of the participants (*N* = 29). About 3% had global developmental delay (*N* = 5). The participants with high body weight (*Z* score > 2.5) were 4 (2.6%). Some participants received medications for these comorbidities or surgical intervention for their congenital heart disease when indicated. Many of them reported stopping medical treatment during the lockdown (88%). All participants received routine compulsory vaccinations. Five cases manifested symptoms and were proved to be COVID-19 patients (3.3%) by polymerase chain reaction test for COVID-19. Most of them were from Giza governorate (4 patients) and 1 was from Cairo. One of them was hospitalized due to COVID-19 complications. All of them received medical treatment for the disease and recovered. There was a case who manifested the symptoms of COVID-19 yet he did not perform the polymerase chain reaction test and was not a COVID-19 patient. His computerized tomography scan revealed unilateral lung abscess for which he received medical treatment and recovered. The number DS cases who did not have COVID-19 yet one or more of their family members had the disease was 4 (2.6%). Some cases manifested sleep disturbances during the lockdown (*N* = 32; 21.3%). Sixty-eight cases exhibited cognitive deterioration as judged by the parents (45.3%). Many cases manifested deterioration of other abilities (e.g. communication or motor abilities) as judged by the parents (*N* = 72; 48%).

**Table 1:** Comparison between groups regarding the survey items, and clinical characteristics

Variables	All cases (*N* = 135)	Group I (Training) (*N* = 33)	Group II (No training) (*N* = 102)	*P* value
*Gender* *(N, %)*				
Male	66 (48.9%)	24 (72.7%)	42 (41.2%)	#0.002*
Female	69 (51.1%)	9 (27.3%)	60 (58.8%)	
Height Z score (mean ± SD)	− 1.14 ± 1.47	− 1.28 ± 1.54	− 1.09 ± 1.45	^0.537
Weight Z score (mean ± SD)	− 0.28 ± 1.25	− 0.32 ± 1.13	− 0.27 ± 1.29	^0.856
Head circumference Z score (mean ± SD)	− 1.16 ± 1.34	− 1.20 ± 1.26	− 1.15 ± 1.36	^0.841
*Care giver’s gender (N, %)*				
Mother	108 (80.0%)	32 (97.0%)	76 (74.5%)	#0.005*
Father/others	27 (20.0%)	1 (3.0%)	26 (25.5%)	
*Care giver’s education (N, %)*				
Low education	68 (50.4%)	23 (69.7%)	45 (44.1%)	#0.011*
Higher education	67 (49.6%)	10 (30.3%)	57 (55.9%)	
*Intellectual disability (Care giver’s opinion) (N, %)*				
No	21 (15.6%)	1 (3.0%)a	20 (19.6%)b	#0.003*
Mild	67 (49.6%)	15 (45.5%)a	52 (51.0%)a	
Moderate	34 (25.2%)	9 (27.3%)a	25 (24.5%)a	
Severe	13 (9.6%)	8 (24.2%)a	5 (4.9%)b	
Comorbidities (*N*, %)	50 (37.0%)	12 (36.4%)	38 (37.3%)	#0.927
COVID-19 (case) (*N*, %)	5 (3.7%)	2 (6.1%)	3 (2.9%)	§0.596
COVID-19 (family) (*N*, %)	4 (3.0%)	2 (6.1%)	2 (2.0%)	§0.251
Sleep disturbances	32 (23.7%)	2 (6.1%)	30 (29.4%)	#0.006*
Cognitive deterioration (*N*, %)	59 (43.7%)	9 (27.3%)	50 (49.0%)	#0.029*
Communication and other abilities deterioration (*N*, %)	72 (53.3%)	11 (33.3%)	61 (59.8%)	#0.008*

Bold indicates the level of significance was taken at *P* value < 0.050

^Independent *t* test. #Chi square test. §Fishers Exact test. *Significant (< 0.050). Homogenous groups had the same symbol (a, b) based on post hoc Bonferroni test

### Comparison between the groups regarding the collected data

The children who were evaluated by Portage program were135 children. Assessment of their anthropometric measures revealed that height, weight, and head circumference Z score ranges were − 3.7 to + 1.9 (− 1 ± 1.4); − 3.1 to + 3.2 (− 0.3 ± 1.3); − 4.8 to 1.5 (− 1.2 ± 1.4) respectively. Despite the fact that certain individuals' physical growth was delayed, there was no significant link between the anthropometric measures and the tested abilities scores. The baseline scores of cognitive, linguistic, and motor abilities ranges were 30–100 (60.7 ± 15.2); 12–100 (46.5 ± 21.5); 13–100 (78.3 ± 15.8) respectively. These 135 cases were divided into two groups. Group I who received virtual home-based training during the pandemic lockdown (*N* = 33;4.4%) and group II who did not receive training (*N* = 102;75.5%). In group I, the percentage of mothers as caregivers was greater than that in group II (97.0% vs. 74.5%). The percentage of parents who did not have higher education was greater in group I (69.7% vs. 44.1%). Group I had a higher percentage of children whose parents thought they had severe ID (24.2% vs. 4.9%). Group I had a lower percentage of people reporting sleep disturbances (6.1% vs. 29.4%), a lower percentage of parents reporting subjective reduction in cognitive abilities of their children during lockdown (27.3% vs. 49%), and a lower percentage of those reporting subjective reduction in communication and other abilities (33.3% vs. 59.8%). The previous data showed significant statistical difference between the groups (Table 1). Comparison between the groups regarding the age of the children, comorbidities, COVID-19 infection frequency and physical growth did not show significant statistical difference.

The objective measurement of the cognitive, language and motor abilities before and after COVID-19 lockdown:

All participants manifested a delay in baseline assessment and a reduction in their abilities after the lockdown regarding all measured domains. The decline in language abilities was the most. The reduction in cognitive abilities was more pronounced in the group I. The reduction in motor abilities was more prominent in the group II (Table 2).

**Table 2:** Comparison between the groups before and after the COVID-19 lockdown regarding the cognitive, linguistic and motor abilities of DS cases

Variables	All cases (*N* = 135)	Group I (Training) (*N* = 33)	Group II (No training) (*N* = 102)	*P* value
*Cognitive abilities score*				
Baseline	60.3 ± 15.0	52.2 ± 13.9	63.0 ± 14.4	^ < 0.001*
After	52.6 ± 14.8	46.3 ± 14.1	54.7 ± 14.5	^0.004*
¤Change	− 7.7 ± 1.8	− 5.9 ± 1.7	− 8.3 ± 1.5	^ < 0.001*
*P* value	¤ < 0.001*	¤ < 0.001*	¤ < 0.001*	
*Language abilities score*				
Baseline	48.9 ± 18.0	49.5 ± 16.8	48.7 ± 18.5	^0.828
After	40.1 ± 18.2	46.0 ± 16.6	40.2 ± 18.3	^0.032*
^†^Change	− 7.8 ± 2.2	− 3.5 ± 1.8	− 8.5 ± 1.7	^ < 0.001*
*P* value	¤ < 0.001*	¤ < 0.001*	¤ < 0.001*	
*Motor abilities score*				
Baseline	78.1 ± 15.2	78.9 ± 14.0	77.9 ± 15.6	^0.722
After	70.5 ± 15.2	73.7 ± 13.8	66.4 ± 15.6	^0.018*
¤Change	− 7.6 ± 2.1	− 5.2 ± 2.2	− 11.4 ± 1.3	^ < 0.001*
*P* value	¤ < 0.001*	¤ < 0.001*	¤ < 0.001*	

Bold indicates the level of significance was taken at *P* value < 0.050

^†^Change = After-baseline (negative values indicate reduction). ^Independent *t* test. ¤Paired *t* test. #Chi square test. *Significant (< 0.050)

Cognitive abilities scores (before and after the lockdown) in group I were lower than those in group II with significant statistical difference. The reduction in cognitive scores in group I was less than that in group II with significant statistical difference. Therefore, the delay in cognitive abilities was less prominent in the group who received home training (− 5.9 ± 1.7 vs. − 8.3 ± 1.5) (Table 2).

The language scores in both groups were reduced after the lockdown yet, the reduction in group I was less than that in group II with significant statistical difference. The language scores after the lockdown were higher in group I than in group II with significant statistical difference. Thus, the delay in language performance was less pronounced in the group who got home-based training (− 3.5 ± 1.8; − 8.5 ± 1.7) (Table 2).

The motor abilities scores after the lockdown in group I were higher than those in group II with significant statistical difference. The motor abilities scores were reduced in both groups after the lockdown. Yet, the reduction in the motor scores in group I was less than that in group II with significant statistical difference. Hence, the delay in motor abilities was less pronounced in the group who performed home-based training (− 5.2 ± 2.2 vs. − 11.4 ± 1.3) (Table 2).

### Logistic regression analysis results

The factors that affected having home-based training were being a mother as a care giver (OR 12.76, 95% CI 1.27 − 127.98), severe ID (caregiver’s opinion) (OR 6.56, 95% CI 1.70 − 25.27), male sex (OR 2.81, 95% CI 1.09 − 7.24), and low education (OR 2.56, 95% CI 1.03 − 6.40) according to logistic regression (Table 3).

**Table 3:** Logistic regression for factors affecting having home-based training

Factors	*β*	SE	*P* value	Odds ratio	
				Value	95% CI
Mother as a care giver	2.55	1.18	0.030*	12.76	1.27 − 127.98
Severe intellectual disability (Care giver’s opinion)	1.88	0.69	0.006*	6.56	1.70 − 25.27
Male sex	1.03	0.48	0.032*	2.81	1.09 − 7.24
Low education	0.94	0.47	0.044*	2.56	1.03 − 6.40
Constant	− 4.82	1.27	< 0.001*		

Bold indicates the level of significance was taken at *P* value < 0.050

*β*: Regression coefficient. SE: Standard error. CI: Confidence interval. *significant (< 0.050)

## Discussion

Individuals with Down syndrome (DS) are considered one of the vulnerable groups to COVID-19 infection. The frequency of COVID-19 in the participants of this study was 3.3%. This was less than the percentage reported by Hüls et al. [12] (83% half of them were hospitalized). Hospitalization risk could be linked to cardiovascular diseases [13]. In this study, the low percentage could be attributed to the young age of the participants and overprotection of their parents. It should be considered that the participants were not recruited from general population, yet from those who were visiting the outpatient clinic for special needs children. This would not allow generalization of the COVID-19 frequency output in DS population and this could be considered a limitation of this study. DS population has high risk for infectious diseases due to genetic susceptibility in some genes related to immunity such as IFNAR1/2 and HSA21 genes and the gene controlling TMPRSS2 receptor. Other factors are involved such as obesity, preexisting comorbidities, hypotonia, and ID which could hinder their healthy habits or social distancing [4, 14, 15]. Therefore, when it comes to DS population, extra precautions should be adopted from families and health care systems to avoid infection and they should have the priority to get COVID-19 vaccine [16].

The lockdown has negatively impacted the health of the participants. Many subjects showed signs of sleep disturbance and a decline in their abilities as rated by their parents. This is consistent with other studies who investigated the lockdown influence on populations other than children with DS. Chang et al. [17] and Mohr et al. [18] reported that containments led to reduced physical activities and excess food consumption, weight gain, and behavioral disorders. The participants in this study manifested variable comorbidities, microcephaly, growth retardation, or obesity which were detected by anthropometric measures. These factors could have shared in exaggerating the unfavorable effect of the lockdown on the participants. Anthropometric measures are one of the main methods used to assess the nutritional condition and the physical growth of children which reflect their health status. Even in absence of microcephaly (standard deviation of head circumference less than − 2.5), low brain volume and reduced connectivity between different brain areas could hinder the development of DS children’s abilities which underscores the necessity of rehabilitation sessions [19, 20].

Cognitive impairment was detected by objective assessment in many participants. This delay which was detected even in the group who got the therapy could be attributed to impaired neurogenesis, and alterations of various neurotransmitters and receptor systems such as serotonin, dopamine, and gamma-aminobutyric acid which was previously reported in such children [21]. Cognitive abilities are not similarly delayed in many individuals with DS. Auditory short-term memory and phonological processing are areas of weakness while visuo-spatial processing, social abilities, and shared attention are generally viewed as relative strengths [22]. Pinter et al. [23] detected a disproportionately small cerebellar volume and a relatively large subcortical gray matter volume in comparison to the overall reduction of brain volume. These characteristics could explain why, despite receiving virtual training, the participants' abilities deteriorated. Because of their reliance on social connection, virtual training may have played a limited role in the present study. Other factors include lack of access to the required materials needed for the continuous training, as well as less time available for the child considering that other children in the household are home-schooling during the pandemic.

The language performance of the participants was the most delayed one. This could be related to expressive language deficits which were reported to surpass the receptive ones. The phonological processing (especially that involving phonological loop) is aberrant which could negatively impact expressive language development, especially morphosyntax. A small hippocampal volume, which is important for memory and other cognitive activities, and a small frontal lobe volume, where the expressive language area is located, could potentially have a role [23, 24]. Childhood apraxia of speech, prosodic dysregulation, disfluencies, and malocclusions have also been described in children with DS, all of which may contribute to reduced speech intelligibility, aggravate their communication difficulty, and result in low language evaluation scale scores [25].

Hypotonia, which can lead to muscle weakness and ligament laxity, could be the reason for the delay in motor abilities despite motor rehabilitation sessions. These motor deficiencies could be linked to slow cerebellar growth and structural changes, as well as craniocervical instability, which is observed in roughly 60% of such children [26]. Furthermore, abnormal collagen structure, which is crucial for muscle integrity, was detected in these people and was linked to aberrant COL6A3 gene signaling [5]. Furthermore, the shape of the pelvic spinal area and hips in people with DS differs, which could disrupt their gait [17]. All these factors could have limited the progress despite having virtual physical therapy rehabilitation sessions.

The factors that could be related to having programs and online sessions at home were investigated. Such factors should be taken into consideration by the health care professionals in order to tackle and overcome the factors that could limit the commitment of the parent to implement home-based training for their children in the future and to improve the efficiency of the virtual online sessions. This is attributed to the fact that this pandemic is spreading in waves which could lead to more lockdowns in the future. Family members play a fundamental role in the intervention programs and need to get support, orientations, and awareness from the health providers to ensure the continuity of treatment and rehabilitation at home [27]. Comparison between group I and group II regarding these possible factors was performed. Caregivers with a low education level may have more time to perform home training considering the possibility of being jobless. Being a male and having severe delay in cognitive abilities as judged by the parents may be motivating factors for the family to continue training at home. On the other hand, sleep disturbances manifested by children could have hindered the family from being committed to persistent training during the lockdown. Kumaravel et al. [28] reported other hazards of COVID-19 pandemic containment such as irritation, and post-traumatic stress. They suggested that these hazards can be reduced by internet-based socializing and working. Parent support, community support, information technological support, and loosening quarantine restrictions are suggestions for solutions for the negative impact of the pandemic on families with special needs children [29]. This support should include mental health support together with financial support required for improving the health care facilities and provide proper parental knowledge about the syndrome and enlightenment of how to be involved in the virtual training programs with minimal effort and how to make the home-based intervention funny instead of a burden on the family and the child.

## Conclusion

COVID-19 pandemic lockdown had a negative impact on the abilities of children with DS, in addition to their wellbeing and mental health. The young age of the participants could have a role in reducing the risk of COVID-19 infection and in limiting its severe progression. The abilities of DS children were reduced even in those who had online sessions. This underscores the importance of direct one-on-one or group sessions during rehabilitation of children with DS to benefit from their social abilities’ strength. Cognitive, language, and motor abilities should be monitored and emphasized during the creation of rehabilitation programs especially virtual home-based ones. Moreover, factors that influenced the parents’ decision to get online training should be tracked and targeted in order to overcome the difficulties and/or the concepts that could prevent families from enduring home-based intervention.

## Data Availability

The datasets used and/or analyzed during the current study are available from the corresponding author on reasonable request.

## Abbreviations

cm: Centimeter
CI: Confidence interval
COL6A3: Collagen type VI alpha 3 chain
COVID-19: Coronavirus disease-2019
DS: Down syndrome
HSA21: Human chromosome 21
IFNAR1/2: Interferon alpha and Beta Receptor Subunit ½
ID: Intellectual disability
IL: Interleukins
Kg: Kilogram
N: Number
SARS-CoV-2: Respiratory syndrome coronavirus 2
SE: Standard error
SPSS: Statistical package for social sciences
TMPRSS2: Transmembrane protease serine 2
WHO: World Health Organization
